# Evaluation of Analysis Approaches for Latent Class Analysis with Auxiliary Linear Growth Model

**DOI:** 10.3389/fpsyg.2018.00130

**Published:** 2018-02-22

**Authors:** Akihito Kamata, Yusuf Kara, Chalie Patarapichayatham, Patrick Lan

**Affiliations:** ^1^Department of Psychology, Department of Education Policy and Leadership, Center on Research and Evaluation, Southern Methodist University, Dallas, TX, United States; ^2^Department of Educational Measurement and Evaluation, Anadolu University, Eskisehir, Turkey; ^3^Simmons School of Education, Southern Methodist University, Dallas, TX, United States

**Keywords:** mixture model, latent class analysis, case-weight approach, one-step approach, three-step approach

## Abstract

This study investigated the performance of three selected approaches to estimating a two-phase mixture model, where the first phase was a two-class latent class analysis model and the second phase was a linear growth model with four time points. The three evaluated methods were (a) one-step approach, (b) three-step approach, and (c) case-weight approach. As a result, some important results were demonstrated. First, the case-weight and three-step approaches demonstrated higher convergence rate than the one-step approach. Second, it was revealed that case-weight and three-step approaches generally did better in correct model selection than the one-step approach. Third, it was revealed that parameters were similarly recovered well by all three approaches for the larger class. However, parameter recovery for the smaller class differed between the three approaches. For example, the case-weight approach produced constantly lower empirical standard errors. However, the estimated standard errors were substantially underestimated by the case-weight and three-step approaches when class separation was low. Also, bias was substantially higher for the case-weight approach than the other two approaches.

## Introduction

Mixture modeling has become a widely used statistical method in behavioral sciences because it allows for an exploration of identification and understanding of latent subpopulations in a given population. Among them, a method where categorical latent trait constructs are identified based on multiple observed categorical variables is specifically referred to as a latent class analysis (LCA) (Lazarsfeld and Henry, 1968; Dayton and Macready, 1998). While identifying and interpreting latent classes may be of the main interest with LCA, researchers may be also interested in how the identified latent classes are related to auxiliary variables, such as covariates and distal outcomes. In other words, researchers are not only interested in latent classes of individuals, but also in potential causes and/or consequences of the class membership (Bakk et al., 2013, 2014). This type of analysis would provide additional information about heterogeneity of the relations, since it is not realistic to assume that all individuals in the population have the same relations to auxiliary variables (Nylund-Gibson et al., 2014). Moreover, researchers may be interested in considering an auxiliary model in conjunction with LCA, such that separate auxiliary model parameters are estimated for each of the latent classes. For example, a simple linear regression model as an auxiliary model to LCA was presented and investigated in Asparouhov and Muthén (2014). In such a modeling, the latent class variable can be thought of a moderator for the auxiliary model (i.e., secondary model). In this paper, this type of a model is referred to as a two-phase mixture model, because the model is consisted of two phases, the LCA model phase and the auxiliary model phase.

One may argue that a single mixture model without a latent class measurement model may be sufficient to describe heterogeneity on the auxiliary model, such as mixture regression and growth mixture model. However, there are contexts where latent classes should be defined by a latent class measurement model, rather than by a single mixture model. For example, Asparouhov and Muthén (2014) and Vermunt (2010) pointed out that a single mixture model approach will not fit a logic of a researcher, if the latent class measurement model is theorized to define latent classes, rather than the mixture distribution of the auxiliary variable or model. In such a case, results from the two-phase mixture model are not necessarily the same as results from the single mixture model. Therefore, it is paramount to identify latent classes by measurement indicators in the latent class measurement model first, rather than directly attempting to identify latent classes based on heterogeneity in their auxiliary variable or model. Thus, an implementation of a two-phase mixture model becomes important.

## Methods to two-phase mixture models

There are several different approaches that can be undertaken to estimate a two-phase mixture model. In this section, four selected approaches are described, although the first approach will not be investigated in this study.

### Classify and analyze approach

Classify-and-analyze approach is a two-step process, also referred to as hard partitioning (Vermunt, 2010). In the first step, LCA is conducted, and each individual is assigned to a specific latent class by the highest posterior class-membership probability that is obtained from the LCA. Then, in the second step, class assignments are used as an observed grouping variable to compare groups on auxiliary variables, if the model contains auxiliary variables. If the model contains auxiliary model, the auxiliary model will be fitted for each of identified classes. In either case, membership in identified classes is mutually exclusive, such that each observation is classified into only one of the identified classes. While it is straightforward to implement (Hibbard et al., 2007; Reinke et al., 2008; Archambault et al., 2009; Hardigan, 2009), this strategy comes with some critical disadvantages. First, there can be misclassified individuals, because deterministic classifications are based on the probabilistic information of class-membership probabilities. It is known that misclassification of individuals in the classify-and-analyze approach can result in biased estimates of the relations between the latent classes and the auxiliary variables and auxiliary model parameters (Hagenaars, 1993; Clogg, 1995). Second, somewhat related to the first disadvantage, classification uncertainties (namely, measurement errors in classifications from the LCA) would be ignored. Since classifications are treated as true states, the standard errors for parameter estimates by the classify-and-analyze approach are likely underestimated (Roeder et al., 1999; Loken, 2004; Clark and Muthén, 2009). Overall, the literature to date is in agreement that the classify-and-analyze approach is no longer recommended for estimating an LCA model with auxiliary variables and/or auxiliary models. Therefore, the classify-and-analyze approach was not considered further in this study.

### One-step approach

The one-step approach involves a simultaneous estimation of an LCA model and auxiliary variables and/or auxiliary models (Formann, 1992; Heijden et al., 1996; Bandeen-Roche et al., 1997; Dayton and Macready, 1998; Muthén and Muthén, 2000; Clark and Muthén, 2009; Kim et al., 2016). The one-step approach is recommended particularly by earlier literature (Heijden et al., 1996; Muthén, 2001), because estimating LCA and auxiliary models in one-step has advantages over the classify-and-analyze approach. First, occurrence of classifying individuals into incorrect classes would be irrelevant, because the one-step approach does not involve classifications of individuals into particular classes based on estimated class probabilities. In other words, the estimation of the latent classes is accomplished jointly by the inclusion of auxiliary variable(s) and/or model(s) (Kim et al., 2016). As underlined by Clark and Muthén (2009), individuals can be fractional members of all identified latent classes in the one-step approach. Thus, it reduces problems that arise from treating the latent classes as a true state, the procedure that is followed by the classify-and-analyze approach. Second, measurement errors of class membership would be incorporated in the analysis, because they are embedded in the model by the one-step approach. Another advantage of the one-step approach is a contribution of the included auxiliary variable(s)/model to the estimation of latent classes. Clark and Muthén (2009) argue that this inclusion improves the class separation and reduces the standard errors.

However, while it is still known as an efficient approach, recent studies are cautious about employing the one-step approach (Vermunt, 2010; Nylund-Gibson et al., 2014). The prominent reason is that the parameters of the first-phase LCA model may be affected by auxiliary variables and/or models, if the strength of the associations between latent class indicators and latent classes are not sufficiently strong (Vermunt, 2010; Asparouhov and Muthén, 2014). If this becomes a problem, it could lead to a different number and/or interpretations of latent classes by including auxiliary variables and/or models. Changing the parameters in this manner would be disconcerting and leads to problems with model construction. While the inclusion of auxiliary variables and/or models is important, the measurement of the latent classes should be free from influence of auxiliary variables and models (Nylund-Gibson et al., 2014).

### Three-step approach

Another approach to a two-phase mixture model is the three-step approach (Bolck et al., 2004; Vermunt, 2010). The key advantage of the three-step approach is a separate treatment of the LCA model and auxiliary variables or models, just like classify-and-analyze approach, while classification measurement errors are still taken into account. As a result, class separation is accomplished without being affected by auxiliary variables and models (Vermunt, 2010; Kim et al., 2016). As the first step with the three-step approach, the LCA model is estimated as a measurement model by using only latent class indicator variables. In the second step, a variable for most likely classes (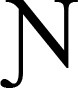) is created by the modal assignment using the largest posterior probabilities obtained in the first step. Just like classify-and-analyze approach, 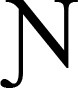 is treated as a manifest nominal variable that represents the class assignments. However, the three-step approach retains the information about classification uncertainties and utilizes it as the measurement errors of classifications as follows. Using the estimated posterior class probabilities and number of the individuals assigned to each of the latent classes, classification uncertainty rates are computed. These rates are the average posterior probabilities in the form of *k* × *k* matrix, where *k* is the number of latent classes. In the third step, the auxiliary model is fit separately for each of the identified classes in the first step by incorporating the measurement errors derived in the second step. Bolck et al. (2004) demonstrated their three-step approach underestimated associations between class membership and auxiliary variables. Vermunt (2010) proposed a correction method by maximizing a weighted log-likelihood function for clustered data. With a series of simulation studies, Vermunt demonstrated that the correction improved the method substantially. Currently, the three-step approach with Vermunt's correction is incorporated in Mplus software (Asparouhov and Muthén, 2014).

Asparouhov and Muthén (2014) demonstrated that the three-step approach with Vermunt's correction recovered parameters very well, when the latent class variable was measured well by the LCA model (i.e., high entropy). Also, it was demonstrated that the loss of efficiency for the three-step approach was minimal, compared to the one-step approach. On the other hand, Bakk et al. (2014) reported that the bias-corrected three-step approach utilized in Mplus software tends to underestimate the standard errors of the auxiliary variables effects. Nylund-Gibson et al. (2014) extended the application of this three-step approach to a latent transition analysis (LTA). Overall, the three-step approach with Vermunt's correction has become a promising method to estimate a mixture model with auxiliary variables and/or auxiliary models. Nonetheless, Asparouhov and Muthén (2014) argued that any method could fail to achieve satisfactory accuracy and efficiency, if the latent class variable is poorly measured by the measurement model (i.e., low entropy), including the three-step approach.

### Case-weight approach

The case-weight approach for mixture models is also a three-step procedure. In the first step, the measurement model (i.e., LCA) is estimated by using only latent class indicator variables. In fact, this first step LCA is exactly the same as the first step of the aforementioned three-step approach. However, how the information about classification uncertainties are derived in the second step is different from the three-step approach. In the second step of the case-weight approach, the estimated posterior class probabilities from the first step are directly saved as weight variables (one weight variable for each identified class). In the third step, the auxiliary model is fit separately for each of the latent classes by using the corresponding weight variable from the second step as the case weights.

This way, each observation is treated as a fractional member of all identified latent classes, as a way to incorporate classification uncertainties. As a result, the contribution of each observation to a given class is represented by the estimated class probability for the observation. For example, if an observation has a very small class probability for a given latent class, the observation will have a very small impact on estimating parameters of an auxiliary model, but not zero. Also, the effective sample size for each class is the sum of the estimated class probabilities, which is a reasonable realization of the estimated class size. This procedure is analogous to computing a weighted data summary quantity, such as a weighted mean, which is also similar to the propensity score weighting procedure (Robins and Rotnitzky, 1995; Hirano and Imbens, 2001).

As one example related to this approach, Clark and Muthén (2009) demonstrated an approach, where the latent class variable was regressed on a predictor variable by using the classification probabilities from the initial-step LCA as regression weights. Cheng (2012) also employed the same approach for an LCA model with a distal outcome. Clark and Muthén, as well as Cheng, confirmed that the weighted regression approach worked well, while the one-step approach was still found to best account for the uncertainty in latent class membership.

The case-weight approach discussed in this paper assumes any kind of latent-class measurement model and any kind of auxiliary model. For example, Nese et al. (2017) employed this approach to study heterogeneity of the growth of emergent literacy knowledge by combining a zero-inflated Poisson regression model (i.e., the latent-class measurement model phase) and a three-class growth mixture model (i.e., the auxiliary model phase). However, the performance of this approach is rather unknown. Thus, the current study aimed to investigate the performance of the case-weight approach under various conditions for a two-phase mixture model through a simulation study. The performance of the case-weight approach was also compared to two other approaches; namely, one-step and three-step approaches.

## Methods

### Model

The first phase of the investigated two-phase mixture model was a two-class LCA model with four dichotomous measurement indicators. The model is expressed as

$$\begin{array}{l} P\left(U_{p}=1|c\right)={\left[1+\exp\left(\tau_{cp}\right)\right]}^{-1}, \end{array}$$

where *U*_*p*_ is the response on the *p*th dichotomous measurement indicator (*p* = 1, …, 4) and *c* is the latent class variable (*c* = 1 or 2). Also, τ_*cp*_ is the threshold parameter for *p*th measurement indicator for latent class *c*. Accordingly, τ_*cp*_ is the logit of *U*_*p*_ = 1, given in the *c*th class.

**Figure 1:**
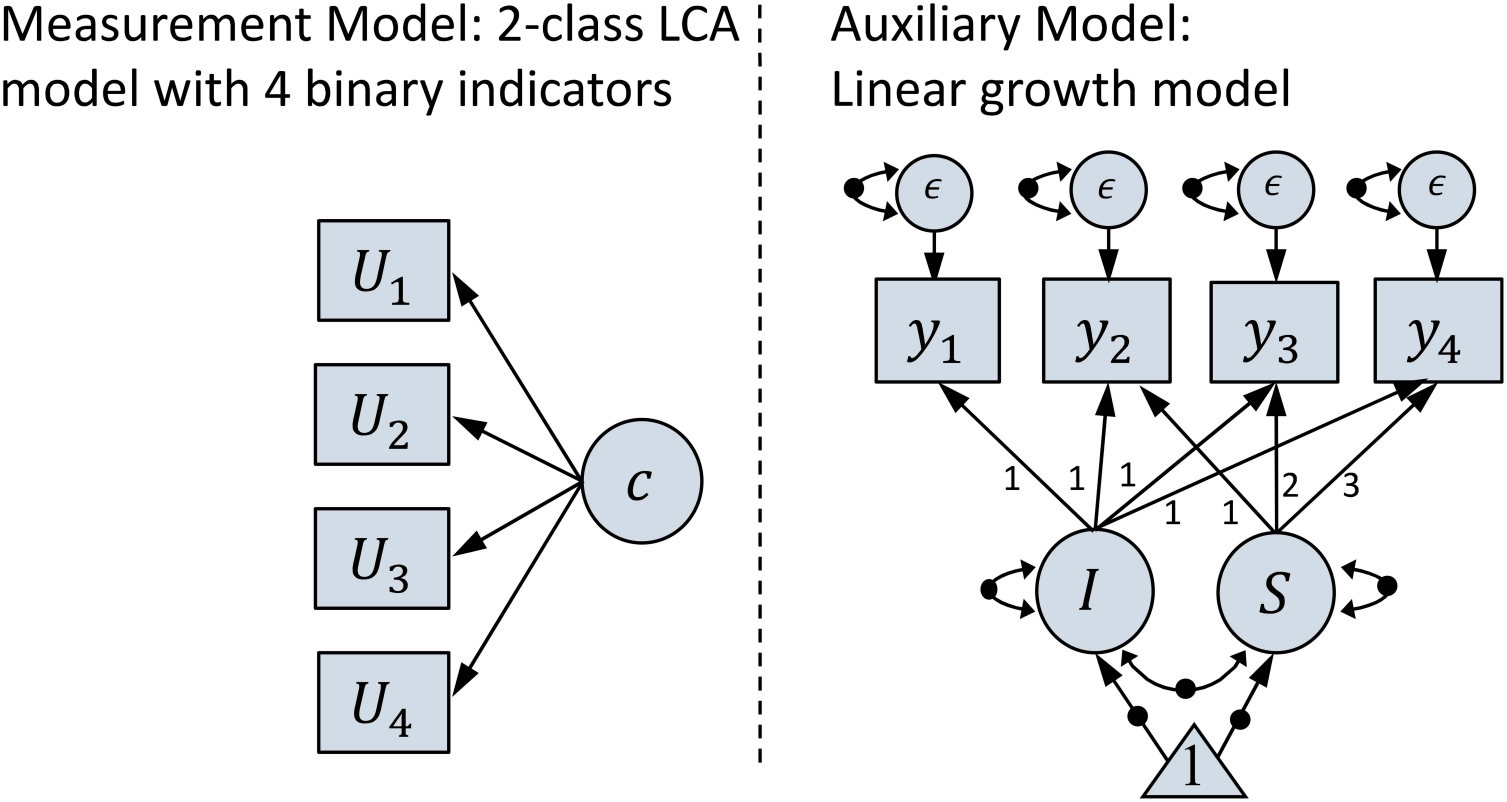
Graphical representation of the studied two-phase mixture model. On the measurement model, *U*_1_, *U*_2_, *U*_3_, and *U*_4_ represent four dichotomous outcome variables related to latent class variable *c*. On the auxiliary model, *y*_1_, *y*_2_, *y*_3_, and *y*_4_ represent repeatedly measured outcome variable at four time points. Also, *I* represents the growth intercept, *S* represents the growth slope, and ϵ represent the residuals. Black dots indicate that parameters represented by these arrows are different between latent classes.

The second phase of the two-phase mixture model was an auxiliary model, which was a linear growth model (LGM) with four time points. The LGM was set up as a special case of a two-factor confirmatory factor analysis model, where the two latent factors represented the growth intercept and growth slope that varied between individuals. The model is expressed as

$$\begin{array}{l} \text{y}=\Lambda \eta +\epsilon , \end{array}$$

where **y** is a 4 × 1 vector of outcome measures, **Λ** is a 4 × 2 matrix of factor loadings, **η** is 2 × 1 vector of two latent factors, and **ϵ** is a 4 × 1 vector of residuals. Factor loadings for the four outcome measures were all constrained to fixed values: [1, 1, 1, 1] for the intercept factor (the first column of **Λ**), and [0, 1, 2, 3] for the slope factor (the second column of **Λ**). As a result, the growth intercept was a realization of the initial status. In addition, **ϵ** was assumed to be normally distributed with 0 means and covariance matrix with equal diagonals and 0 off-diagonals, indicating that error variances for the four outcome measures were constrained to be equal and zero covariances between errors. In addition, **η** was assumed to be normally distributed with unknown means (mean intercept and mean growth trajectory) and covariance matrix (variances of intercept and growth trajectory, and covariance between intercept and growth trajectory). All parameters in the auxiliary model (i.e., mean intercept, mean slope, intercept variance, slope variance, covariance between intercept and slope, and error variances) were assumed to be different between the two latent classes. A graphical representation of this two-phase mixture model is also provided in Figure 1. As mentioned above, all parameters in the auxiliary model were assumed to be different between latent classes. These parameters are graphically indicated as dots on straight and curved arrows in Figure 1.

The true parameter values for the LCA model were varied, including the class proportion for the smaller class. Hereafter, the smaller class will be referred to as “class 2.” The threshold parameters were constrained to be the same for the four measurement indicators, but the value was varied depending on simulation conditions (see below). These differences in threshold parameter values indirectly affected differences in class separation (i.e., entropy), where a lower threshold resulted into a lower class separation. Parameters for the auxiliary LGM were assumed to be different between the two classes, but fixed for all simulation conditions. The parameter values for the auxiliary model are provided in Table 1. Note that we did not hypothesize any direct relations between the auxiliary model variables and latent class indicators, just like in Bakk et al. (2013).

**Table 1:** True parameter values for the auxiliary model.

**Parameter**	**Class 1 (larger class)**	**Class 2 (smaller class)**
Mean(*I*)	0.6	0.4
Mean(*S*)	1.0	1.8
Variance(*I*)	1.9	1.4
Variance(*S*)	0.4	0.3
Covariance(*I, S*)	0.5	0.3
Variance(ϵ)	0.5	0.7

*I, intercept; S, slope; ϵ, residuals*.

### Simulation study

Data were generated for the two model phases simultaneously, just like how Asparouhov and Muthén (2014) generated data. According to Asparouhov and Muthén, this data generation strategy generates data that would be consistent with a 2-phase mixture model, because the latent class variable is not an endogenous variable in the data generation model. Data sets were generated for a total of 27 within-method simulation conditions, with a minimum of 1,000 replications for each condition. We generated additional replications if there were fewer than 1,000 successfully converged replications that correctly identified the 2-class model as the best model by BIC for any of the analysis methods. We followed this strategy only for fitting the 2-class model, because the parameter recovery evaluations were undertaken only when the 2-class model was fitted. In addition, if any methods that had more than 1,000 successfully converged replications with 2-class model as the best model by BIC for a particular condition, only the first 1,000 replications were evaluated for parameter recovery evaluations.

The 27 within-method simulation conditions were represented by three simulation factors; namely, sample sizes, class proportion for the smaller class (class 2), and class separation (i.e., threshold parameter in the LCA phase of the model). These three simulation factors were chosen, because they are known to affect the performance of mixture model estimation. Three sample sizes were: small (500 examinees), medium (1,000 examinees), and large (2,000 examinees). Three class-2 proportions were: small (0.05), medium (0.15), and large (0.30). Note that this study generated latent classes only by a two-class LCA model. Lastly, three levels of class separation (the threshold parameter the LCA phase of the model) were: low (0.754), medium (1.254), and high (1.750). These threshold parameter values were computed by first defining the log-odds difference between classes for the LCA phase of the model; low = 1.50, medium = 2.50, and high = 3.50. As a result, the average entropy was 0.66, 0.77, and 0.90 for the three levels of the class separation in the simulation. Data generated for each of the 27 within-method simulation conditions were fitted by three methods, namely, one-step approach (OS), case-weight approach (CW), and three-step approach (TS).

For each simulation condition, the model fit for the 2-class model was evaluated relative to 1-class and 3-class models. To do so, the proportion of replications, in which Bayesian information criterion (BIC) for the 2-class model was smaller than ones for 1-class and 3-class models, was computed for each of the three methods for each of the 27 within-method simulation conditions. For the case-weight and three-step approaches, this evaluation was commonly performed for the first-step LCA model, because it would be the step where one would make a model selection decision regarding the number of latent classes for these two approaches. Also, convergence rate was evaluated for the 1-class, 2-class, and 3-class models for each simulation condition. Note that a computation of the convergence rate for the CW and TS approaches involved a multiplication of the convergence rate of the first-step LCA model and the convergence rate of the third-step auxiliary LGM model.

Finally, parameter recovery performance was evaluated for the 2-class model, separately for the three approaches for each auxiliary model parameter for the two latent classes for each of the 27 within-method simulation conditions, by computing; (a) absolute relative bias, (b) empirical standard error (SE), (c) the mean estimated SE relative to the empirical SE, and (d) root mean square error (RMSE). Then, each of the four indices were averaged across all model parameters for the two latent classes separately for each of the 27 within-method simulation conditions. As mentioned earlier, only the first 1,000 successfully converged replications were included in the parameter recovery evaluations, including only replications that concluded the 2-class model was correctly selected by the BIC.

Note that a bias is the systematic part of the estimation error. In this study an absolute relative bias was computed by taking the absolute value of a relative bias value (i.e., bias divided by the true parameter value). For a given parameter θ ,

$$\begin{array}{l} {\left(absolute\;relative\;bias\right)}_{\theta }=\left|\frac{\left(\frac{{\sum^{\text{​}}}_{i=1}^{r}{\hat{\theta }}_{i}}{r}\right)-\theta }{\theta }\right|, \end{array}$$

where ${\hat{\theta }}_{i}$ is the parameter estimate for the *i*th replication, θ is the true parameter value, and *r* is the number of replications. On the other hand, an empirical SE is the random part of estimation error that attributes to sampling and was computed as the standard deviation of repeatedly obtained 1,000 parameter estimates for a given parameter θ by

$$\begin{array}{l} {\left(empirical\;SE\right)}_{\theta }=\sqrt{\frac{\sum_{i=1}^{r}{\left({\hat{\theta }}_{i}-\left(\frac{\sum_{i=1}^{r}{\hat{\theta }}_{i}}{r}\right)\right)}^{2}}{r}}, \end{array}$$

where all symbols are defined above. Also, each simulation replication produced an estimated SE, and it is explicitly referred to as the “estimated SE” in this study to distinguish it from the empirical SE. The empirical SE is a numerically realized theoretical SE based on repeatedly sampled data, while the estimated SE is an analytically (or numerically, in some other cases, such as the bootstrap method) estimated SE based on one given sample data. In practice, only an estimated SE will be available to data analysts and will be treated as the best estimate of the theoretical SE. Therefore, it would be of interest how much the estimated SEs are close to the theoretical SE (i.e., the empirical SE) to evaluate the quality of the estimated SEs. Therefore, the mean of the estimated SEs was computed across 1,000 replications, and its magnitude was compared to the empirical SE by their ratio to evaluate potential under- or over-estimation of the estimated SEs. Finally, RMSE is the total estimation error, and it was computed for a given parameter θ by

$$\begin{array}{l} {\left(RMSE\right)}_{\theta }=\sqrt{\frac{\sum_{i=1}^{r}{\left({\hat{\theta }}_{i}-\theta \right)}^{2}}{r}}, \end{array}$$

where all symbols are defined above.

Mplus software (Muthén and Muthén, 1998–2012) was used to generate the data, as well as to fit the model. Data generations and analyses with Mplus were controlled by R software (R Core Team, 2016). Examples of Mplus syntax are provided as a Supplementary Material.

## Results

### Convergence rate

Convergence rates are summarized in Table 2. Although they are not shown in the table, all replications converged without any warning or error for the 1-class one-step approach and first step 1-class LCA model. Also, almost all replications of the first-step 2-class LCA model converged, which was shared by the case-weight and three-step approaches, with the lowest convergence rate of 97.1%.

**Table 2:** Percentages of convergence and correct model selection.

**Sample size**	**Class-2 proportion**	**Class separation**	**Convergence: OS approach**		**Convergence: CW approach**		**Convergence: TS approach**		**Correct model selection**	
			**2-Class**	**3-Class**	**2-Class**	**3-Class**	**2-Class**	**3-Class**	**OS**	**LCA**
		Low	29.4	2.7	96.9	80.1	50.0	32.7	11.3	7.1
	Small	Medium	64.5	5.8	98.9	72.7	71.1	37.0	61.3	86.0
		High	80.5	7.3	97.5	62.4	81.2	38.7	79.7	99.5
		Low	83.3	6.9	99.4	78.3	84.5	40.1	81.2	70.4
*n* = 500	Medium	Medium	94.8	9.1	100.0	53.8	94.5	38.1	93.9	99.8
		High	98.4	9.3	100.0	51.4	97.9	42.1	97.9	99.8
		Low	98.3	8.8	100.0	75.5	97.4	49.9	97.2	97.8
	Large	Medium	99.9	11.7	100.0	47.5	99.5	41.2	98.9	100.0
		High	99.9	10.5	100.0	53.6	99.9	48.3	98.6	99.6
		Low	53.2	5.2	96.8	74.8	51.4	26.3	24.2	9.2
	Small	Medium	84.6	9.4	99.7	62.9	85.5	27.4	84.5	98.1
		High	93.7	11.9	99.8	48.0	94.6	31.4	93.7	100.0
		Low	96.1	10.4	99.9	67.8	94.6	33.9	96.0	94.2
*n* = 1,000	Medium	Medium	99.4	11.3	100.0	41.9	99.2	28.8	99.4	100.0
		High	99.9	11.2	100.0	36.0	99.9	25.9	99.9	100.0
		Low	99.9	11.9	100.0	55.5	99.9	35.0	99.8	99.7
	Large	Medium	100.0	11.2	100.0	35.3	100.0	29.2	99.9	100.0
		High	100.0	12.3	100.0	44.7	100.0	37.7	100.0	100.0
		Low	79.6	8.8	98.0	69.1	56.9	15.3	61.2	19.8
	Small	Medium	96.2	12.3	100.0	53.1	95.1	20.5	96.2	100.0
		High	98.6	15.3	100.0	38.2	98.7	19.4	98.6	100.0
		Low	99.7	11.3	100.0	55.5	99.3	20.3	99.7	100.0
*n* = 2,000	Medium	Medium	100.0	12.8	100.0	31.6	100.0	18.1	100.0	100.0
		High	100.0	12.7	100.0	33.9	100.0	20.9	100.0	100.0
		Low	100.0	11.8	100.0	36.7	100.0	22.9	100.0	100.0
	Large	Medium	100.0	12.8	100.0	31.2	100.0	23.3	100.0	100.0
		High	100.0	15.3	100.0	40.7	100.0	31.9	100.0	100.0

*OS, one-step approach; CW, case-weight approach, and TS, three-step approach. LCA was common first step for CW and TS approaches*.

For the 2-class model, the case-weight approach had the highest convergence rate among the three methods. For example, they converged nearly 100% for all conditions when *n* = 2,000, while its convergence rate dropped somewhat when the class-2 proportion was small with *n* = 500. Nonetheless, its convergence rates were always higher than 96%. The convergence rates for the three-step approach had a similar pattern as the case-weight approach, namely, when class-2 proportion was small, convergence rate was lower. However, the convergence rates were constantly lower than the ones for the case-weight approach within the same conditions. In some conditions, they were substantially lower, especially when *n* = 500, and/or when the class-2 proportion was small. Even with *n* = 2,000, when the class-2 proportion was small and the class separation was low, the convergence rate dropped to 56.9%, whereas the convergence rate remained nearly 100% for the case-weight approach. On the other hand, the convergence rate for the one-step approach dropped to even lower percentages with lower sample size, smaller class-2 proportion, and/or lower class separation. For example, the convergence rate was 79.6% when the class-2 proportion was small and the class separation was low even with *n* = 2,000. It dropped to only 29.4% in the same condition with *n* = 500.

For the 3-class model, convergence rates for the one-step approach dramatically dropped. The highest convergence rate was only 15.3% for the conditions with *n* = 2,000 and high class separation. On the other hand, the convergence rates remained high for the case-weight approach, although they were uniformly lower than 2-class model in comparable conditions. For the three-step approach, convergence rates for 3-class model dropped much more than the case-weight approach. Yet, convergence rates were considerably higher than the ones for the one-step approach.

### Model selection

Percentages of correct model selection are summarized in the last two columns of Table 2. First, correct model selection rates were quite low either by the one-step approach or the first-step LCA when class separation was low and class-2 proportion was small. For this combination of the conditions, correct model selection rates were always low, regardless of the sample size.

On the other hand, correct model selection rates were 100% or nearly 100% with high class separation and large or medium class-2 proportion, regardless of the analysis method and the sample size. Also, conditions with medium class separation and large class-2 proportion demonstrated quite high correct model selection rates. With high class separation and small class-2 proportion, the correct model selection rate was nearly 100% with *n* = 2,000 (98.6% for OS and 100% for first-step LCA). However, the rates decreased as the sample size became smaller for OS; 93.7% with *n* = 1,000, and 79.7% with *n* = 500, while the rates remained near 100% for the first-step LCA. Similar patterns were observed for conditions with medium class separation and medium class-2 proportion.

Overall, the first-step LCA (i.e., case-weight approach and three-step approach) was better in correct model selection than the one-step approach. Exceptions were when class separation was low and class-2 proportion was small. Another exception was when class separation was low and class-2 proportion was medium with *n* = 500.

### Parameter recovery

**Figure 2:**
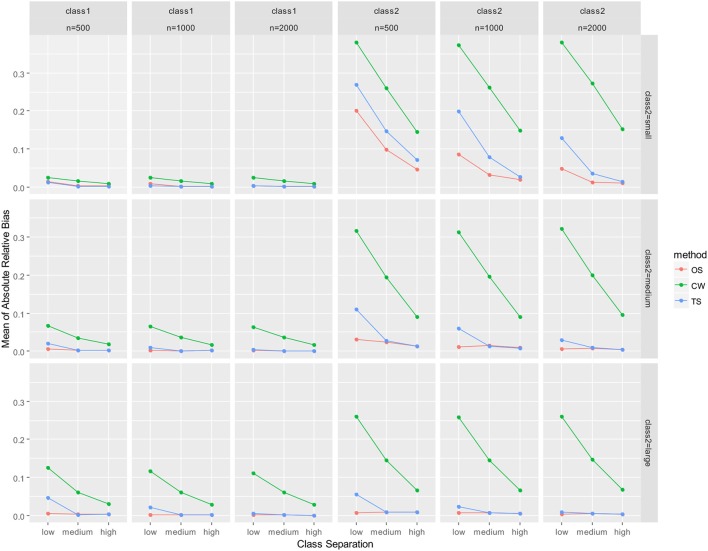
Averaged absolute relative bias for auxiliary model parameters. OS, one-step approach; CW, case-weight approach, and TS, three-step approach.

**Figure 3:**
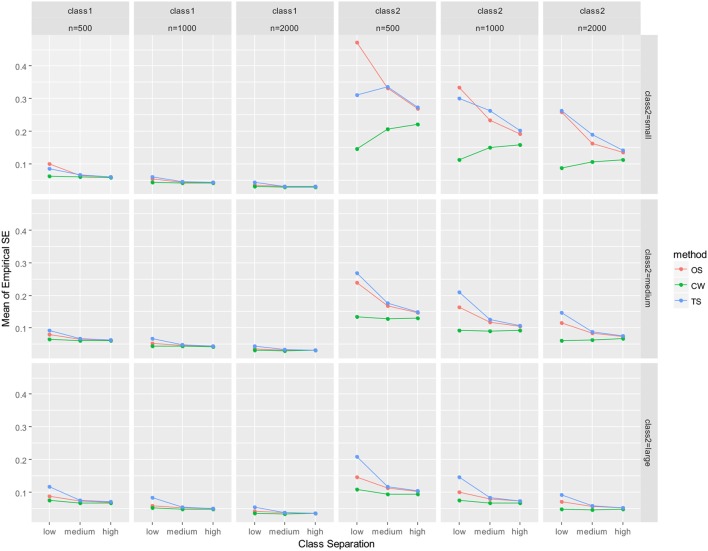
Averaged empirical SE for auxiliary model parameters. OS, one-step approach; CW, case-weight approach, and TS, three-step approach.

**Figure 4:**
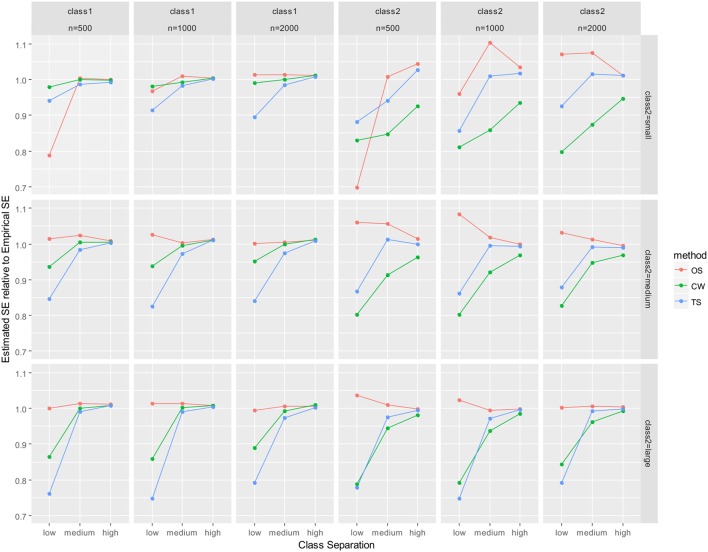
Averaged mean estimated SE relative to mean empirical SE for auxiliary model parameters. OS, one-step approach; CW, case-weight approach, and TS, three-step approach.

**Figure 5:**
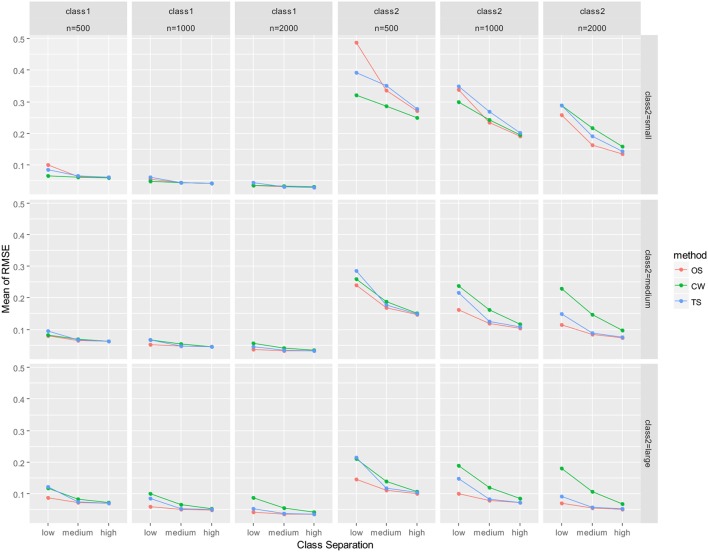
Averaged RMSE for auxiliary model parameters. OS, one-step approach; CW, case-weight approach, and TS, three-step approach.

As mentioned earlier, parameter recovery results were summarized by averaging for all parameters in the auxiliary LGM for each latent class. The summary results are presented in Figure 2 (mean of absolute relative bias), Figure 3 (mean of empirical SE), Figure 4 (mean of estimated SE relative to empirical SE), and Figure 5 (mean of RMSE). For each figure, results are summarized into three columns of graphs for three sample sizes (*n* = 500; *n* = 1,000; *n* = 2,000) for each latent class. The first three columns of graphs are for the larger class (class 1), and the last three columns of graphs are for class 2 (smaller class). Three rows of graphs are for the three levels of the class-2 proportion (small; medium; large). The three ticks on the horizontal axis of each graph are three levels of class separation (low; medium; high).

For the larger class (class 1), all of absolute relative bias, empirical SE, and RMSE were substantially smaller. Particularly, differences between the three approaches were nearly undistinguishable for class 1 for high class-separation conditions, regardless of sample size and class-2 proportion. The only exception was the relative estimated SE, where underestimation of the estimated SE was revealed for the case-weight and three-step approaches, especially when class separation was low. Underestimation of estimated SE was nearly zero for conditions with medium or high class separation for all three approaches. Interestingly, underestimation was much larger by the one-step approach than the other two approaches when class separation was low, class-2 proportion was small, and *n* = 500.

There were some important observations for results for the smaller class (class 2). Hereafter, discussions of the results are focused on class 2. First, it was revealed that the mean of absolute relative bias (Figure 2) was larger for the case-weight approach than the other two approaches in all conditions. Relative bias for the one-step approach and the three-step approach sharply decreased as the sample size became larger, as the class separation became higher, and as the class-2 proportion became larger. However, relative bias for the case-weight approach was affected much less by the class-2 proportion and the sample size, while it was still affected by the class separation. In other words, larger sample size and larger class-2 proportion did not reduce the relative bias by the case-weight approach. On the other hand, relative bias for all three approaches decreased sharply as the class separation became higher, and the discrepancy between the case-weight approach and the other two approaches became smaller when the class separation was high. Overall, the one-step and three-step approaches displayed strength with respect to relative bias, while the case-weight approach did not.

Although details are not presented in this paper, results for each parameter were examined under *n* = 500 conditions. The mean and variance parameters of the slope for class 2 was particularly high in relative bias by all three approaches when the class-2 proportion was small and the class separation was low. However, sharp decrease was observed for all three approaches as the class separation became higher. Also, sharp decrease was observed for the one-step and three-step approaches as the class-2 proportion became larger. Overall, it was confirmed that relative bias for the case-weight approach was constantly higher than the two other approaches for all parameters for the smaller class. Also, it was confirmed that the discrepancy between the three approaches became smaller as the class separation became higher.

With respect to empirical SE (Figure 3), the performance of the case-weight approach was better than the other two approaches, especially when the class-2 proportion and the sample size was small. However, the discrepancies between the three approaches became smaller as the class separation became higher and the class-2 proportion became larger. The performance of the one-step and three-step approaches were similar; when the class separations were medium or high, their empirical SEs were nearly identical, especially under medium and large class-2 proportion conditions. Overall, the case-weight approach displayed strength with respect to empirical SE. To evaluate potential under- or over-estimation of the estimated SE, the relative magnitude of the mean estimated SE to empirical SE was evaluated (Figure 4). As a result, the case-weight and three-step approaches displayed substantial underestimation of the SE for both classes particularly when class separation was low. For class-1 parameters, underestimation for the two approaches became small when class separation was medium or high. However, for class-2 parameters, underestimation for the case-weight approach did not diminished under small class-2 proportion conditions. Another notable result for the underestimation of the estimated SE was that the one-step approach displayed substantial underestimation for both class-1 and-2 parameters under the most demanding condition (*n* = 500, small class-2 proportion, and low separation) compared to the case-weight and three-step approaches.

As empirical SEs were evaluated for each parameter for *n* = 500 conditions (again, details are not presented here), they were notably high for the mean and variance of the intercept for class 2 by the one-step and three-step approaches when the class separation was small and the class-2 proportion was small. As the class-2 proportion became larger, empirical SE values improved for the one-step and three-step approaches, however, empirical SE values were still constantly lower by the case-weight approach.

With respect to RMSE (Figure 5), the performance of the one-step and three-step approaches were nearly identical and slightly better than the case-weight approach under medium/high class separation and medium/large class-2 proportion conditions. When class-2 proportion was small, the one-step approach performed slightly better than the three-step approach for larger sample sizes (*n* = 1,000 and 2,000). The case-weight approach performed better than the other two approaches in limited conditions. First, under small class-2 proportion conditions with *n* = 500, the case-weight approach performed constantly better than the other two approaches. Also, the case-weight approach performed better than the other two approaches under small class-2 proportion and low class separation condition with *n* = 1,000.

When RMSE were evaluated for each parameter for *n* = 500 conditions (again, not presented here), they were constantly low for class-1 parameters for all three approaches. For class-2 parameters, the case-weight approach constantly performed better than the other two approaches for three parameters; latent factor covariance (i.e., covariance between intercept and slope), the mean and variance of the intercept. However, the case-weight approach constantly performed worse than the other two approaches for the mean of the slope.

## Conclusions

This study investigated the performance of three selected approaches for estimating two-phase mixture model, where the first phase was a two-class LCA model and the second phase was a LGM with four time points. There were some important observations in relation to the literature. First, according to Asparouhov and Muthén (2014), the loss of efficiency for the three-step approach would be minimal, compared to the one-step approach. Our results confirmed that this was the case. On the other hand, according to Asparouhov and Muthén (2014) and Vermunt (2010), parameters of the LCA model may be affected by auxiliary models, if the strength of the associations between the latent class indicators and latent classes are not sufficiently strong. This made us anticipate that parameter recovery for one-step approach would suffer in conditions with low class separations. Also, it was our hope that the case-weight approach and/or three-step approach would show better results than the one-step approach. However, it was not the case with respect to bias. One-step approach was less affected by low class separation. Also, our results displayed substantial underestimation of estimated SE for the case-weight and three-step approaches in certain conditions, which is consistent with Clark and Muthén (2009), Vermunt (2010) and Bakk et al. (2014).

## Practical implications

Some practically important results were demonstrated in this study. First, it was revealed that case-weight approach displayed constantly larger bias than the other two approaches. It should be noted that this is a critical limitation of the case-weight approach. On the other hand, one-step and three-step approaches displayed much smaller bias. Their bias values were nearly identical especially when class separation was medium or high. However, their biases were high, when class-2 proportion was small, class separation was low, and the sample size was not large. Second, it was found that the case-weight approach had a strength with respect to empirical SE. However, one should be cautioned that estimated SEs were quite underestimated by the case-weight approach. Also, correct model selection rates were extremely low in such demanding conditions for all approaches, including the case-weight approach. Therefore, in practice one may not be able to take advantage of the strength of the case-weight approach with respect to SE, because there will be a lot of uncertainty in correct model selection in such demanding conditions.

Regarding successful convergence, it was found that one-step approach was very sensitive to demanding conditions. Practically, this will make one-step approach difficult to use unless the data are from ideal conditions, such as large sample size, medium to high class separation, and no presence of small class proportion. On the other hand, convergence rate was a strength of the case-weight approach under the demanding conditions. This strength makes case-weight approach allow one to explore and test more model options even in less ideal conditions. However, the case-weight approach should be used with caution in practice, because it come with substantially larger bias than the other two approaches.

Based on the results of this study, our recommendation for an application of a two-phase mixture model is as follows. First, ensure that the sample size is sufficiently large, a minimum of 500, as Asparouhov and Muthén (2014) and Vermunt (2010) have already suggested. Second, fit the latent-class measurement model part by itself to explore the number of latent classes. This makes sense because this study has demonstrated that the first-step LCA would identify a correct model better than the one-step approach. Also in this stage, it is recommended to ensure (a) the class separation is reasonably high, such as entropy >0.80, (b) there is no small class with <15%, to utilize the three-step approach. If these two conditions are not met, or sample size is not as large as 2,000, it is recommended to implement the one-step approach. However, if these conditions become more challenging (lower class separation and presence of smaller class), the one-step approach and the three-step approach may not converge. If so, it is when the case-weight approach is recommended to be fit. However, even if the case-weight approach converges, the results should be used with caution.

## Limitations

The investigated model in this study was limited to a very specific model. As mentioned earlier in this paper, the case-weight approach and three-step approach can be applied to any kind of latent-class measurement model and any kind of auxiliary model. For example, Nese et al. (2017) employed this approach to study heterogeneity of the growth of emergent literacy knowledge by combining a two-class zero-inflated Poisson regression model as the latent-class measurement model phase, and a three-class growth mixture model as the auxiliary model phase. A future study to investigate the performance of the one-step, case-weight and three-step approaches in such a complex model is warranted.

## Author contributions

All authors listed have made a substantial, direct and intellectual contribution to the work, and approved it for publication.

### Conflict of interest statement

The authors declare that the research was conducted in the absence of any commercial or financial relationships that could be construed as a potential conflict of interest.
