# Supplementary material for: Evaluation of Analysis Approaches for Latent Class Analysis with Auxiliary Linear Growth Model
Source: Front Psychol. 2018 Feb 22;9:130. doi: 10.3389/fpsyg.2018.00130 (PMC5826956; doi:10.3389/fpsyg.2018.00130)
Supplement: Supplementary file 1 [file DataSheet1.docx]

Appendix

Examples of Mplus Syntax

Here we present examples of the Mplus syntax we used to generate data and fit the models with studied approaches. As examples, all syntax below are for 2-class models. For the data generation syntax, it is particularly for one of the 27 within-method conditions, in which *n* = 500, class-2 proportion = large, and class separation = high.

**Data Generation**

Montecarlo:

Names are u1-u4 y1-y4;

Generate = u1-u4(1);

Categorical = u1-u4;

Genclasses = c(2);

Classes = c(2);

Nobservations =500;

Nrep = 1;

save=tempdata.dat;

Analysis: Type = Mixture;

Model Population:

%Overall%

i s | y1@0 y2@1 y3@2 y4@3;

y1-y4*0.5 (1);

[c#1@0.8472979];

%c#1%

[u1$1*1.750374];

[u2$1*1.750374];

[u3$1*-1.750374];

[u4$1*-1.750374];

i with s*0.5;

[i*0.6];

[s*1];

i*1.9;

s*0.4;

y1-y4*0.5;

%c#2%

[u1$1*-1.750374];

[u2$1*-1.750374];

[u3$1*1.750374];

[u4$1*1.750374];

i with s*0.3;

[i*0.4];

[s*1.8];

i*1.4;

s*0.3;

y1-y4*0.7;

**Step 1 for Case-weight and 3-step Approaches**

DATA: FILE IS mydata.dat;

VARIABLE: NAMES ARE u1-u4 y1-y4;

USEVARIABLES = u1-u4;

CLASSES = c (2);

CATEGORICAL = u1-u4;

AUXILIARY = y1-y4;

ANALYSIS: TYPE = MIXTURE;

STARTS = 100 20;

SAVEDATA: SAVE=CPROB;

FILE=step1_cls2.dat;

**Step 3 for Case-weight Approach**

DATA: FILE IS step1_cls2.dat;

VARIABLE: NAMES ARE u1-u4 y1-y4 p1 p2 n;

USEVARIABLES = y1-y4 p1 p2;

TRAINING = p1 p2 (PROBABILITIES);

CLASSES = c (2);

ANALYSIS: TYPE = MIXTURE;

STARTS =100 20;

MODEL:

%overall%

i s | y1@0 y2@1 y3@2 y4@3;

y1-y4 (1);

%c#1%

i-s;

i with s;

y1-y4 (2);

%c#2%

i-s;

i with s;

y1-y4(3);

**Step 3 for Three-step Approach**

DATA: FILE IS step1_cls2.dat;

VARIABLE: NAMES ARE u1-u4 y1-y4 p1 p2 n;

USEVARIABLES = y1-y4 n;

CLASSES = c (2);

NOMINAL = n;

ANALYSIS: TYPE = MIXTURE;

STARTS = 100 20;

MODEL:

%overall%

i s | y1@0 y2@1 y3@2 y4@3;

y1-y4 (1);

%c#1%

[n#1@4.814]; !This value is read from Step 1 output.

i-s;

i with s;

y1-y4 (2);

%c#2%

[n#1@-1.938]; !This value is read from Step 1 output.

i-s;

i with s;

y1-y4 (3);

**One-step Approach**

DATA: FILE IS mydata.dat;

VARIABLE: NAMES ARE u1-u4 y1-y4;

USEVARIABLES = u1-u4 y1-y4;

CLASSES = c (2);

categorical = u1-u4;

ANALYSIS: TYPE = MIXTURE;

STARTS = 100 20;

MODEL:

%overall%

i s | y1@0 y2@1 y3@2 y4@3;

y1-y4 (1);

%c#1%

i-s;

i with s;

y1-y4 (2);

%c#2%

i-s;

i with s;

y1-y4(3);
